# Eccentric strengthening vs. conventional therapy in sub-acute stroke survivors: a randomized controlled trial

**DOI:** 10.3389/fneur.2024.1398860

**Published:** 2025-01-23

**Authors:** Kalthoum Belghith, Mustapha Zidi, Lhéo Vincent, Jean-Michel Fedele, Rayan Bou-Serhal, Wael Maktouf

**Affiliations:** ^1^Bioengineering, Tissues and Neuroplasticity, UR 7377, Faculty of Health/EPISEN, University of Paris-Est Créteil, Créteil, France; ^2^EMEIS Group, Clinique du Parc de Belleville, Paris, France

**Keywords:** muscle strength, stiffness, ankle joint, eccentric training, stroke

## Abstract

Spastic paresis, a frequent consequence of stroke, is characterized by both neurological and muscular alterations, leading to decreased muscle strength, increased passive muscle stiffness, and subsequently, diminished functional capacity. Although conventional rehabilitation programs are effective in enhancing muscle strength, they often fail to yield clinically significant improvements in functional capacities. Eccentric Training (ET) has shown promise in addressing the shortened muscle fascicle lengths and joint contractures commonly observed in stroke survivors. Despite the prevalence of contractures and rigidity in this population, the effects of ET on the structural and mechanical properties of muscles remain underexplored. This study aims to investigate the impact of ET on gait speed in sub-acute stroke patients compared to conventional therapy. Additionally, we aim to explore the effects of ET on the mechanical properties, structural characteristics, and neuromuscular parameters of the plantar flexors. A randomized controlled trial will be conducted, adhering to CONSORT guidelines, with participants assigned to either a Conventional Therapy Group or an Eccentric Training Group. Assessments will be conducted at baseline, and after ET intervention, encompassing clinical, biomechanical, and functional evaluations. This study seeks to provide empirical evidence on the efficacy of ET in improving motor outcomes in sub-acute stroke patients, thereby informing more effective rehabilitation strategies.

## 1 Introduction

Stroke, as the leading cause of adult disability, presents a substantial challenge for affected individuals (1). Among the primary impairments following a stroke is the weakening of the lower limb muscles (2), impacting over 90% of stroke patients (3). While some recovery of lower limb function is observed, it often falls short of enabling independent and comfortable outdoor ambulation in daily life (4). Consequently, approximately half of stroke survivors are unable to resume their professional activities, and roughly two-thirds experience chronic disability (5).

One of the primary neuromotor consequences of a stroke is spastic paresis, known as the most common motor disorder following a cerebral injury (6). Generally, the spastic paresis syndrome is associated with both neurological and muscular disorders (7). Neurological issues manifest immediately after a stroke, leading to a quantitative reduction in the recruitment of motor units in agonist muscles (8). This has been explained by a failure of central voluntary control activation and changes in the structure and properties of spinal motoneurons (9). These alterations lead to a decrease in muscle contraction efficiency, resulting in a diminished capacity to generate voluntary force (10). In addition to the previously mentioned neurological alterations, a stroke also triggers muscular changes (11). These alterations, referred to as spastic myopathy, result from a combination of two factors: the underuse of the paretic limb and immobilization in a shortened position (12). Spastic myopathy induces multiple adaptations in the mechanical and structural properties of the paretic muscle (2, 13). In this context, studies have demonstrated a loss of fascicular length and thickness of plantar flexors (PF) (14) and a decrease in pennation angle of the gastrocnemius medialis (GM) in stroke survivors (15). Regarding mechanical adaptation, stroke injury leads to muscle contracture, which is defined as an increased muscle stiffness during passive mobilization of the ankle joint (14, 16).

Several studies have investigated many rehabilitation programs for stroke survivors and found that a regular musculotendinous stretching is effective in short-term spasticity inhibition and in preventing long-term hypo-extensibility and muscle contracture; muscle strengthening is recommended as soon as voluntary motor control allows for it; relearning grasping (17), balance (18), and locomotion involves the repetition of motor tasks (19). Otherwise, many studies have concluded, with a high level of evidence, the effectiveness of botulinum toxin in reducing spasticity (20). However, motor improvement remains mild to moderate (21). These mixed results concerning motor function improvement are likely related to the evaluation methods of movement-limiting factors and muscle hyperactivity, which are lengthy, intrusive, and destructive (3, 13). They also do not focus on reducing the effects of spastic myopathy, which is still not well-understood in management (22, 23). Therefore, it is both justified and necessary to question the post-stroke rehabilitation and readaptation methodologies.

Recently, an increasing amount of recent research is focusing on muscle strengthening, particularly eccentric training (ET) (24). ET, a traditional method used to boost muscle strength in athletes, involves muscle contraction during the lengthening of the musculotendinous complex (25). Compared to other training modalities at the same force exertion level, eccentric exercise requires less muscle activity (26). Therefore, this approach could be particularly effective for addressing muscle fascicle shortening and stiffness observed in stroke survivors, due to the increased mechanical load involved during active lengthening (14). This increased load is hypothesized to lead to more significant gains in muscle strength and volume and may also reduce hyperactivity in affected muscles (24). In this context, ET has been demonstrated to enhance muscle strength, reduce spasticity, and improve functional performance and quality of life in chronic stroke survivors following 4 to 6 weeks of intervention (27, 28). As a result, ET offers a myriad of benefits that may be relevant for individuals with neurological conditions (27). However, to our knowledge, no study has investigated the effects of ET on the structural and mechanical properties of the PF, nor on their implications for improving functional capacities. This is particularly relevant given the high prevalence of stiffness in post-stroke patients, which is associated with alterations in the structural and mechanical properties of the muscle-tendon complex, consequently limiting functional capacities (29). These issues are challenging to address in neurorehabilitation, where conventional therapy often fail to produce clinically significant results (30, 31). In fact, conventional therapy, which generally involves stretching exercises, concentric training, and repetitive movements to relearn skills such as balance and locomotion, does not appear to significantly improve the patient’s functional walking abilities beyond 0.04 m/s (32).

The objectives of this study are: (i) investigating the effects of eccentric training on the structural and mechanical properties of PF in stroke survivors compared to conventional therapy, and (ii) analyzing the relationships between changes in structural and mechanical parameters of PF and improvements in walking speed and maximal range of motion.

## 2 Materials and methods

### 2.1 Ethical approval and trial registration

The study protocol, patient information letter, and informed consent form received Institutional Ethics Committee approval (CPP 2022–038 = 000117). The study is registered in the ClinicalTrials.gov database (ID = NCT06140381). The procedures will be conducted according to the principles expressed in the Declaration of Helsinki. Written consent to participate in the protocol will be signed directly by the patient.

### 2.2 Study design

**Figure 1:**
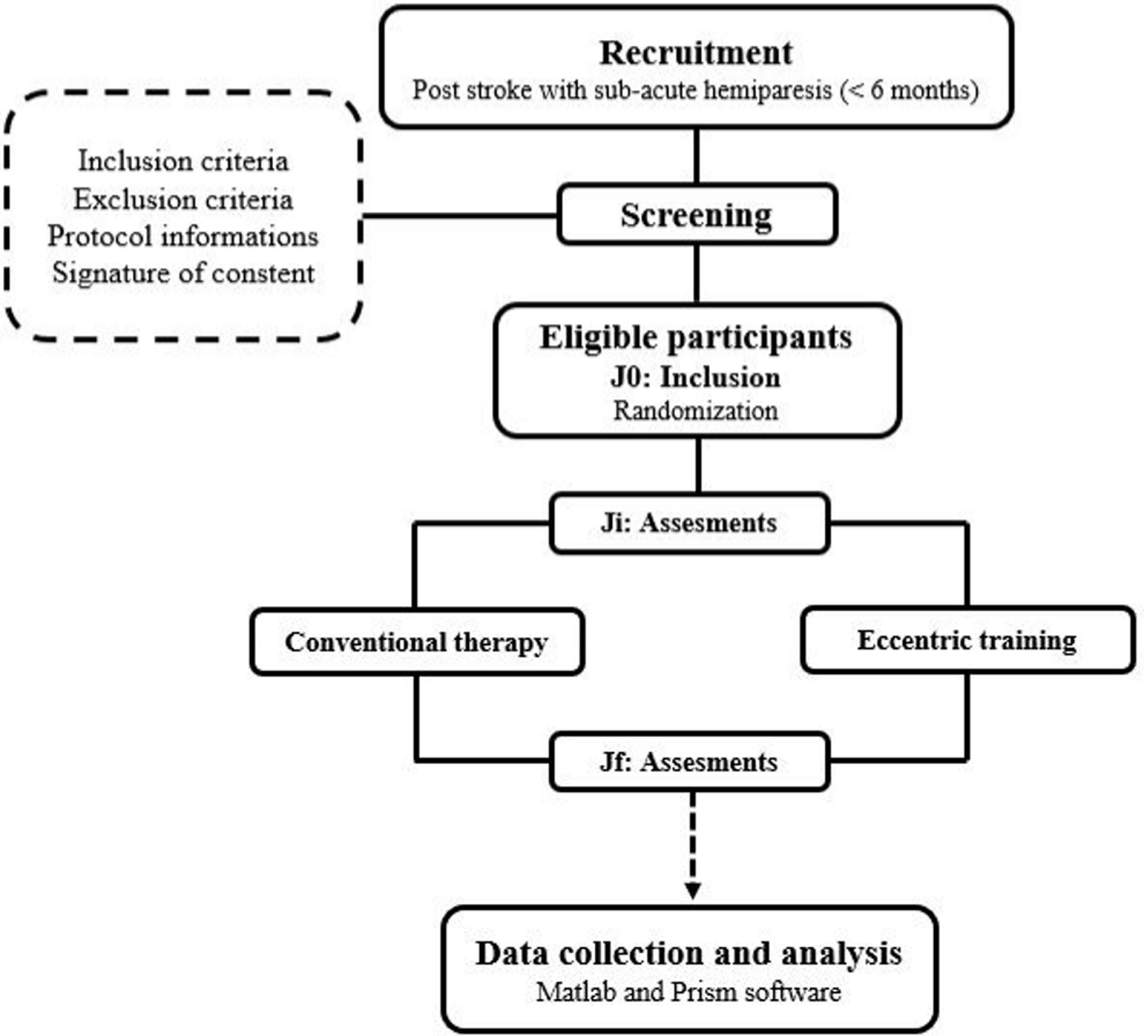
Study design.

This study will be conducted in strict adherence to the Consolidated Standards of Reporting Trials (CONSORT) guidelines, and will employ a single-blinded, controlled, randomized design, wherein participants will be randomly assigned to one of two groups: the conventional therapy group (CTG) or the Eccentric training group (ETG). Both groups will undergo a series of assessments at: Ji (initial assessment) and Jf (final assessment) as illustrated in Figure 1. The study will begin with a recruitment phase, followed by a screening phase. Subsequently, there will be a 4-week period allocated for experimental testing before the intervention, and an additional 4 weeks for experimental testing after the intervention. The experimental testing will include clinical health assessments, biomechanical assessments, neuromuscular evaluations, and functional assessments.

### 2.3 Randomization

The random assignment of participants to their respective groups will take place on the day they will be included in the study and will be supervised by the investigator. The randomization list will be created by an independent statistician from the clinical research unit. For the randomization process, a computer-generated list will be generated using the Clinical Trial Randomization Tool. This list will then be uploaded into an online case report form. Each study participant will be assigned a unique allocation study number in a sequential format (TMP00X). The rigorous blinding will be maintained until the database will be finalized. A blinded assessor will conduct three visits: one before, one in the middle, and one after the interventions, while unblinded kinesiologists, who provide treatment and exercise sessions, will not be involved in the assessment sessions. All required information stipulated by the study protocol will be entered diligently into the Electronic Data Capture system in real-time as it will be acquired.

### 2.4 Recruitment

Participants will be recruited from the clinic of Parc de Belleville in Paris between the 1st of January 2024 to 31st May 2024. Recruitment will be accomplished by disseminating study participation offers in clinical centers, and subsequently, the medical staff will compile a list of volunteers to the experimenter. To participate in this study, subjects will be invited for an admission consultation (Pre-inclusion) (J0) with the principal investigator. Before this consultation, the investigator will need to ensure that the patient meets the study’s inclusion and exclusion criteria.

#### 2.4.1 Inclusion criteria

- Adult aged 20 to 75 years.
- Subacute hemiparesis (< 6 months).
- BMI between 18.5 and 25.
- Written consent to participate in the study.

#### 2.4.2 Exclusion criteria

- Ankle impairment
- Botulinum toxin injections in PF within the last 4 months before study inclusion to ensure that the muscle properties were not influenced by the toxin’s effects.
- Medical contraindication for maximal effort.
- Neurodegenerative disorders.
- Cardiovascular disorders.
- History of epilepsy.

The investigator will explain the purpose of the study, and the experimental protocol. The patient will then be given time for consideration. If they will agree, on the day of admission, they will sign an informed written consent to participate in the study, and then they will undergo their initial evaluation (Ji).

### 2.5 Intervention

#### 2.5.1 Conventional therapy

The conventional therapy program will extend over a period of 12 weeks, consisting of a total of 36 sessions with three sessions per week conducted by physiotherapists. The program will include both isometric and concentric strengthening exercises specifically targeting the plantar flexors. The load and intensity of these exercises will be individualized according to each participant’s capabilities. The conventional therapy will consist of:

- Isometric Exercises: Participants will perform isometric contractions at 70% of their MVC for 10 s, followed by a 20 s rest interval. This cycle will be repeated in sets of 10.
- Concentric Exercises: These exercises will involve the use of weights or resistance bands. The intensity of these exercises will progressively increase from 50 to 80% of the MVC over the course of the program. The number of repetitions per set will also gradually increase from 8 to 12, with each exercise being performed in three sets.
- Stretching Sessions: The program will incorporate stretching exercises for the plantar flexors, performed both passively and actively. Each stretch will be held for 30 s and repeated three times for each target muscle.

#### 2.5.2 Eccentric training protocol

**Figure 2:**
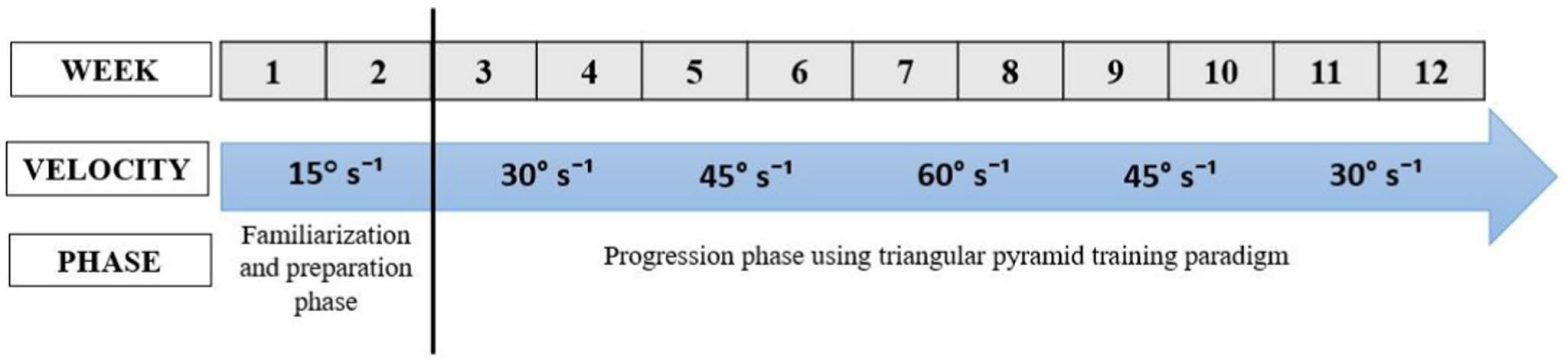
The progression of eccentric training protocol.

In the absence of a standardized eccentric isokinetic training protocol established for hemiparetic subjects on the plantar flexor muscles, the protocol designed for this study drew inspiration from the one established by Clark and Patten (28) and by Harris-Love et al. (33). This protocol will allow the introduction of eccentric stimulus to individuals who are new to this type of training. It will progressively advance their program to include workload levels that are sufficient to stimulate muscular plasticity optimally, thereby inducing skeletal muscle adaptations. The ET protocol will adhere to guidelines provided by the TiDieR, which encompass the following 12 items:

1. *Name:* The ET protocol.
2. *Why:* The ET protocol will aim to enhance walking capacity and reduce spasticity.
3. *What (Materials):* The eccentric exercise program will be carried out using the BIODEX isokinetic device (Biodex 4 Medical Systems, Inc., Shirley, NY, USA).
4. *What (Procedures):* The eccentric program will last for 12 weeks, with a frequency of 3 sessions per week, totaling 36 sessions. At least 1 day of rest will be required between two training sessions. The participants will be placed in the similar position of the neuromuscular assessments (reference position). The Biodex System 4 dynamometer settings for the exercise sessions will be conducted under settings like those used for the assessments. The training program will be divided into two phases: a familiarization phase and a progression phase. Training will be progressing according to the schedule presented in the Figure 2. The familiarization phase, involving the manipulation of three key variables, will aim to reduce risks and enhance the advantages of eccentric strength training. This phase will unfold in two stages (33). The first stage will consist of two sessions designed to familiarize participants with the muscle recruitment patterns associated with isokinetic eccentric exercises. The second stage will span a one-week preparation period, intended to elicit a protective muscular response, and progressively condition the muscles for higher eccentric intensities (33). For the progression phase, a triangular pyramid training paradigm will be employed (28). The rationale will be to, first, train to increase the speed at which force can be produced and second increase the load produced during dynamic contractions. Custom-designed attachments will be used to enable optimal biomechanical alignment for proper performance of the ankle joint movement (28). Participants will be instructed to “resist and try to stop the dynamometer.” Verbal encouragement will continue to be provided throughout the training sessions to motivate maximal effort. This form of training will conceptually remain like power training, which involves rapid muscle contractions. Power training will continue to be considered a safe but intense form of training that is more effective than traditional resistance training using slower contractions.
5. *Who:* By a kinesiologists specialized in adapted physical activity.
6. *How:* Face to face sessions.
7. *Where:* Rehabilitation department.
8. *When and how much:* the ET protocol begins the 1st of January 2024 and concludes on the 31^st of^ May 2024. Each week will include three sessions, making a cumulative total of 36 sessions. Each session lasts 45 min, with a 15-min warm-up and 30 min of actual exercise.
9. *Tailoring:* The determination of the number of sets will be tailored to the patient’s abilities.
10. *Modifications:* Modifications will be implemented to the protocol in each session, involving adjustments such as an increase in the number of sets or repetitions.
11. *How well (planned):* The training program will be divided into two phases: a familiarization phase and a progression phase. Training will be progressing according to the schedule presented in the Figure 2. The program’s progression will be determined by two factors: a quantitative element linked to the training load (volume/intensity) and a qualitative aspect associated with the nature of the exercises (eccentric exercise).
12. *How well (actual):* Not started yet.

### 2.6 Outcome measures

#### 2.6.1 Functional parameters

First, gait speed (m/s) will be measured through the 10-meter walking test. Secondly, the maximal range of motion (ROM), defined as the extent of stretch that the participant can comfortably endure during the stretching maneuver, will be measured using the BIODEX isokinetic device (Biodex 4 Medical Systems, Inc., Shirley, NY, United States). Participants will be positioned prone on the isokinetic machine and instructed to achieve complete relaxation. To minimize the involvement of unrecorded muscles, the ankle will be securely fixed in a relaxed position on the isokinetic dynamometer. Starting from the neutral position (0°), the ankle joint will be passively mobilized from plantar to dorsiflexion at a slow angular velocity (2°/s). Participants will be encouraged to maintain a state of relaxation and halt ankle rotation when they will perceive a tolerable maximum stretch. Then, the ROM (°) will be recorded for each participant.

#### 2.6.2 Biomechanical proprieties of plantar flexors

Muscle stiffness, as expressed by the shear modulus (*μ*, in Kpa), will be assessed during passive ankle joint mobilization and during MVC of PF (34). An Aixplorer^®^ ultrasound scanner (Supersonic Imagine, version 6.1, Aix-en-Provence, France) will be used in Shear Wave Elastography (SWE) mode (musculoskeletal preset, penetration mode, scale: 600 kPa) to quantify muscle stiffness in PF (GM, GL, and SOL). During the evaluations, two ultrasound probes will be used: probe 1 (4–15 MHz, SL15-4; SuperSonic Imagine, Aix-en-Provence, France) for the GM and GL, and probe 2 (2–10 MHz, SL10-2; SuperSonic Imagine, Aix-en-Provence, France) for the SOL. Acoustic gel will be used as an interface between the skin and the probe. For passive muscle stiffness evaluation, three positions will be identified: P0 = 0° (Neutral position), P1 = 10° and P2 = 20° (from neutral position to dorsiflexion). For each position, the SWE measurement will be conducted twice in predetermined regions, with the order randomized and a 1-min rest period between measurements. Regarding the active stiffness evaluation, the reference position will require the hips to be flexed at 45°, the knee on the paretic side to be fully extended, and the ankle on the paretic side to be positioned at 90°. The ankle joint’s axis will align with the dynamometer’s axis of rotation, and the foot will be securely fastened to the dynamometer platform. To ensure stability, additional attachments will be employed to secure the thigh and trunk to the isokinetic ergometer seat. Then, participants will receive instructions to perform two maximal isometric contractions (MVC) of PF. For submaximal contractions (30, 50, 70% of MVC), participants will receive immediate visual feedback on their %MVC torque to achieve the experimenter’s target %MVC torque. Once the desired torque level will be reached, an ultrasound image will be taken. For each MVC torque level, the SWE measurement will be performed twice in each muscle in a randomized order. Each trial will have a maximum duration of 5 s, and subjects will be allowed rest periods between trials. SWE measurement during passive and active conditions will be used for further analysis.

#### 2.6.3 Structural parameters

Fascicle length, thickness and pennation angle will be assessed using the ultrasound scanner (Supersonic Imagine, version 6.1, Aix-en-Provence, France) in B-mode (echography mode). Muscle thickness will be determined as the distance between the superficial and deep aponeuroses of the GM muscle (35). Fascicle length will be calculated by extrapolating the intersection with both aponeuroses and measuring the distance between their respective intersection points (35). Pennation angle will be defined as the angle between the fascicle and the deep aponeurosis (35). Fascicle length and pennation angle will be measured for three fibers, and the average will be used for analysis. For each parameter, the average of three measurements will be used for further analysis.

#### 2.6.4 Neuromuscular parameters

Peak force and rate of force development (RFD) will be evaluated using the BIODEX isokinetic device (Biodex 4 Medical Systems, Inc., Shirley, NY, United States) during MVC of PF. The peak force value from the two trials of MVC will be recorded (aPeak, N). The relative peak force (rPeak, N/kg) will be calculated by normalizing the peak force to the participant’s body mass (aPeak/BM, N/kg). The dynamometer signals will be stored offline for subsequent analysis. Early RFD will also be obtained from each MVC contraction onset to 50 ms (RFD50-100). Late RFD will be acquired from 100 to 200 ms (RFD100-200). All RFD will be calculated from the linear slop of the force – time curve (*Δ* force/Δ time). Finally, maximal activation will be evaluated using the Trigno Wireless Electromyography (EMG) system (Delsys, Inc., Boston, United States) during the MVC of PF. Four EMG sensors will be placed on the GM, GL, SOL, and tibialis anterior (TA) muscles. Before attaching the electrodes, the skin will be carefully prepared by shaving and cleaning it with an abrasive cleaner and alcohol swab to minimize impedance. EMG sensors will be positioned on each muscle’s belly, aligned parallel to the muscle fibers as recommended by SENIAM guidelines (36). The placement of EMG electrodes will be meticulously verified using ultrasound to ensure longitudinal alignment with the muscle fascicles and proper positioning away from neighboring muscles (37). EMG signal will also be recorded during passive evaluation.

### 2.7 Data analysis

Data will be processed using MATLAB software (MATLAB R2024a, MathWorks, Natick, USA). Ultrasound images will be exported from Aixplorer’s software. Shear displacements will be calculated using a speckle-tracking algorithm. Tissue displacement maps will be used to calculate shear-wave velocity (SWV, m/s) in each pixel of the map. Then, the shear modulus (*μ*) will be calculated as follows:


$$\mu =\rho .SWV^{2},\mathrm{where}\;\rho \;\mathrm{is}\;\mathrm{the}\;\mathrm{muscle}\;\mathrm{mass}\;\mathrm{density}\;\left(1,000\;\mathrm{kg}/m^{3}\right)\text{.}$$

Image processing will be converted each pixel of the color map into a shear modulus based on the recorded color scale. Mean shear modulus values will be calculated in a 15 × 15 mm^2^ region of interest in different regions of each muscle fascicular area (38).

The raw EMG signals will be band-pass filtered at 15–500 Hz through a second-order Butterworth digital filter to remove noise or movement interference (39). The data from the different assessments will be collected, rectified, and smoothed using root mean square analysis (RMS) with a 20–ms window (40) calculated using the following equation (36):


$$\sqrt{\frac{1}{T}}\overset{t0+T/2}{\underset{T0-T/2}{\int }}{\left(EMG\right)}^{2}dt,\mathrm{where}\;T\;\mathrm{is}\;\mathrm{the}\;\mathrm{Time}\;\mathrm{of}\;\mathrm{integration}$$

For the MVC assessments, a moving window with a width of 20 ms will be used to find the maximum RMS EMG activity resulting from the three efforts of MVC for each kind of contraction. Then, all RMS EMG data from the different tests will be normalized using the following equation for each muscle:


$$EMG\;RMS\%=\left[\left(RMS\;EMG\;\mathrm{assessment}/RMS\;EMG\;MVC\right)\;x\;100\%\right]\text{.}$$

### 2.8 Statistical methods

Statistical analysis will be conducted using Prism 7.0 software (GraphPad Software, Inc., San Diego, United States). The normality of data distribution will be assessed using Kolmogorov–Smirnov tests. If the data follows a normal distribution, the paired t-test (for comparing the same group before and after intervention) and independent t-test (for comparing the control and experimental groups) will be employed. If the data does not follow a normal distribution, the Wilcoxon signed-rank test (paired samples) and the Mann–Whitney U test (independent samples) will be applied. Relationships between different parameters and their variations will be evaluated using Pearson’s correlation analysis. Data will be presented by their means and standard deviations. The chosen significance threshold will be set at *p* < 0.05 for all results.

## 3 Discussion

To the best of our knowledge, this study protocol will be the first to investigate the effects of eccentric training on the structural and mechanical properties of PF in stroke survivors compared to conventional therapy. We hypothesize that a notable distinction will emerge between the two interventions, with the protocol incorporating eccentric strengthening demonstrating favorable outcomes. Moreover, the enhancement in gait speed and ROM will be associated with an improvement in passive and active PF stiffness.

The conceptualized program for this study aims to provide high-intensity training for individuals in sub-acute phase to effectively stimulate various forms of plasticity. The speed, load, and number of repetitions are the factors that form the basis of the eccentric work and upon which its relevance depends. Therefore, these parameters will be considered to achieve the targeted objective. In this context, a recent study conducted by Le Sant et al. (24) has presented compelling evidence that ET significantly enhances motor performance, particularly in terms of maximal strength and power, among individuals within neurological populations. This finding aligns with the observations of Clark and Patten (28) suggesting that the remarkable strength improvements attributed to eccentric training post-stroke may be linked to heightened quadriceps stretch reflex activity. This increased reflex activity supplements the voluntary neural drive during eccentric knee flexion contractions. Conversely, when considering the structural properties of the paretic muscle, (41) calculated the minimum detectable change for the thickness parameter, estimating it to be at 3 mm. Remarkably, these authors observed a noteworthy increase of 3 mm in the thickness of the SOL muscle following a 23-session eccentric training program focused on the PF, but this research was conducted with healthy individuals.

The conceptualized program for this study aims to provide high-intensity training for individuals in the sub-acute phase to effectively stimulate various forms of plasticity. The key factors—speed, load, and number of repetitions—form the basis of eccentric work and are critical to its efficacy. Therefore, these factors will be meticulously considered to achieve the targeted objectives. In this context, a recent systematic review by Le Sant et al. (24) presents compelling evidence that ET could significantly enhance motor performance, particularly in terms of maximal strength and power, among individuals with neurological diseases. The remarkable strength improvements attributed to ET post-stroke may be linked to heightened stretch reflex activity (28). Increased reflex activity may supplement the voluntary neural drive during eccentric contractions. Furthermore, regarding the structural properties of the paretic muscle, Geremia et al. (41) observed noteworthy increases of fascicle lengths (GM: 13.2%; GL: 8.8%; SOL: 21%) and muscle thickness (GM: 14.9%; GL: 15.3%; SOL: 19.1%) following 12 weeks ET focused on the PF in healthy individuals. We hypothesize that similar structural enhancements could be observed in post-stroke individuals after ET.

In our recent study, we demonstrated that the paretic muscles exhibited greater stiffness in comparison to the healthy muscles and the highest stiffness was predominantly observed in the GM, especially in the distal region at 20° (42). These observations prompt considerations regarding the potential relevance of eccentric exercise in the context of paretic muscle. In fact, eccentric exercise involves active stretching of the muscle, thereby engaging both the contractile and elastic components of the skeletal muscle. Notably, studies involving ultrasound imaging in healthy individuals have shown that this type of training can induce a reduction in passive muscle stiffness (43, 44), an increase in fascicle length, and muscle thickness (45, 46). These adaptations are likely the result of an increase in the number of sarcomeres in series and in parallel (47). Such muscle adaptations contribute to enhanced muscular strength, particularly at greater muscle lengths (48), by improving the tension-length relationship of the muscle (48, 49). Moreover, ET, which involves lengthening the muscle while producing force, is particularly effective in the context of stroke rehabilitation. It can increase force production, reduce muscle stiffness, and consequently improve the overall function of the muscle-tendon complex and functional capacity (45, 50, 51). However, to the best of our knowledge, no studies have thoroughly examined the potential modifications or improvements in the biomechanical properties of the paretic muscle following eccentric exercise. This gap in research limits our ability to draw conclusive insights into the potential enhancements or lack thereof in addressing the inherent issues associated with spastic paresis.

Walking performance is influenced by lower-extremity muscle strength (28). Particularly, the PF are pivotal in the walking process, serving as agonists and contributing to concentric activity that significantly impacts the quality of propulsion during walking (52). However, the effect of ET interventions on walking function in individuals with spastic paresis has produced mixed results in prior studies. While some investigations have reported significant improvements in walking speed and function (53, 54), others have not observed significant enhancements (55). This variability in outcomes may be attributed to the timing of intervention, as some studies were conducted during the chronic phase, typically after six months post-injury, when spasticity may have become fully established. This study seeks to address this potential limitation by implementing the intervention during the sub-acute phase. Furthermore, other factors such as the intensity of eccentric exercise, its duration, and the overall methodology of intervention may also play a role in these divergent findings, warranting further investigation.

## 4 Limitations

One notable limitation of this study is the heterogeneity within the stroke patient population. Even though participants are in the sub-acute phase, the variability in individual recovery trajectories, severity of stroke, and pre-existing health conditions could lead to significant differences in response to the interventions. This heterogeneity may influence the outcomes and interpretations, making it challenging to draw definitive conclusions that are broadly applicable to all sub-acute stroke patients. We aim to increase the sample size to minimize the effects of data heterogeneity and interpretation bias. Furthermore, this study will focus on penniform muscles. It has been suggested that SWV measurements with small deviations from the fiber direction, as determined by the probe-fascicle angle, may be potentially inaccurate (56). However, previous study on the GM have shown relatively reproducible stiffness measurements (57).

## 5 Conclusion

This study protocol proposes an innovative comparison between ET and conventional therapy for improving outcomes in post-stroke survivors during the sub-acute phase. Preliminary evidence supports ET’s potential to enhance structural, neuromuscular, biomechanical, and functional parameters, with notable improvements expected in muscle strength, stiffness, and walking performance. However, existing research has yet to fully elucidate the specific biomechanical adaptations in paretic muscles following ET, highlighting a significant gap in our understanding. This study aims to address this gap, offering new insights into stroke rehabilitation practices. The outcomes of this research could inform future clinical approaches, emphasizing the critical need for high-quality, targeted interventions in the early stages of stroke recovery.
